# Plasma Level of MMP-10 May Be a Prognostic Marker in Early Stages of Breast Cancer

**DOI:** 10.3390/jcm9124122

**Published:** 2020-12-21

**Authors:** Barbara Maria Piskór, Andrzej Przylipiak, Emilia Dąbrowska, Iwona Sidorkiewicz, Marek Niczyporuk, Maciej Szmitkowski, Sławomir Ławicki

**Affiliations:** 1 Department of Aesthetic Medicine, Medical University of Bialystok, 15-267 Bialystok, Poland; andrzej.przylipiak@umb.edu.pl (A.P.); emila_lubowicka@wp.pl (E.D.); niczy.ma@gmail.com (M.N.); 2 Clinical Research Centre, Medical University of Bialystok, 15-276 Bialystok, Poland; iwona.sidorkiewicz@gmail.com; 3 Department of Biochemical Diagnostics, Medical University of Bialystok, 15-269 Bialystok, Poland; msz@umb.edu.pl; 4 Department of Population Medicine and Civilization Diseases Prevention, Medical University of Bialystok, 15-269 Bialystok, Poland; slawicki@umb.edu.pl

**Keywords:** stromelysins, MMP-3, MMP-10, plasma concentration

## Abstract

Background: Stromelysins are potential breast cancer biomarkers. The aim of the study was to evaluate if plasma levels of selected metalloproteinases (MMPs) (stromelysin-1 (MMP-3) and stromelysin-10 (MMP-10)) and cancer antigen 15-3 (CA 15-3) used separately and in combination demonstrated diagnostic usefulness in breast cancer (BC). Methods: The study group consisted of 120 patients with BC, while the control group included 40 patients with benign breast cancer and 40 healthy individuals. Concentrations of MMP-3 and MMP-10 were determined by enzyme-linked immunosorbent assay; CA 15-3 was determined by chemiluminescent microparticle immunoassay. Results: In the group of patients with BC, the area under the curve (AUC) was significantly higher for all markers (except MMP-3) and all sets of markers. At the earliest disease stage, only MMP-10 had a significantly higher AUC (AUC = 0.8692, *p* < 0.001). Moreover, MMP-10 had the highest AUC (0.9166) among parameters tested separately. The highest AUC was observed for the combination of MMP-10 + CA 15-3 and MMP-3 + MMP-10 + CA 15-3 in line with disease progression (stage I 0.8884 and 0.8906, stage II 0.9244 and 0.9308, stages III + IV 0.9919 and 0.9944, respectively, *p* < 0.001 in all cases). Conclusions: The results suggest that MMP-10 could be a potential marker in early stages of BC. Moreover, plasma concentration of MMP-10 and MMP-3 in combination with CA 15-3 may improve diagnosis of this type of cancer.

## 1. Introduction

Stromelysin-1 (MMP-3) and stromelysin-2 (MMP-10) are members of the family of metalloproteinases (MMPs), which are zinc-dependent enzymes. These stromelysins have similar substrate activity, for example, ability to cleave extracellular matrix (ECM) proteins such as aggrecan, fibronectin, and collagen types III and IV. Active forms of MMP-3 and MMP-10 share 78% identity in amino acid sequence. The stromelysins demonstrate differences in optimal pH, enzymatic activity, and expression in different cells [1]. MMP-3 is expressed both in healthy cells, for example, endothelial cells, epithelial cells, stromal fibroblasts and macrophages, and cancerous cells [2]. Expression of MMP-10 has not been detected in normal tissues. Interestingly, MMP-10 is expressed in infections, in response to injury and in cancer transformation [3].

Cancer is a major public health problem and the second leading cause of death in Poland and also worldwide [4]. Breast cancer (BC) is the most common malignant tumor among women [5]. It is estimated that 5000 out of every 15,000 patients newly diagnosed with BC in Poland will not survive [6]. At present, biomedical research provides extensive knowledge of signaling pathways connected with cancer development, molecular transformations in tumor cells, and cancer microenvironment. Interplay among these three factors plays a crucial role in cancer progression and metastasis. Despite considerable research efforts, the role of MMPs in carcinogenesis has not yet been elucidated. As mentioned above, MMPs are critical to ECM degradation. However, they are also able to degrade the basement membrane, which leads to the initiation of cancer metastasis [7]. The spread of cancer cells to surrounding lymph nodes and tissues, and subsequently distant organs is the main cause of cancer mortality. Understanding molecular mechanisms at the cellular level and changes occurring in the surrounding microenvironment can contribute to the development of new diagnostic and treatment strategies. It is believed that the epithelial–mesenchymal transition (EMT) is a critical moment in the regulation of epithelial cell morphology and function [8]. Molecular changes associated with EMT include impairment of cell and cell-matrix adhesion, cleavage of basal membrane components, and ECM degradation caused by alterations in MMPs activity. Hypoxia and acidification in the tumor microenvironment induce MMPs synthesis in cancer and stromal cells. EMT enables malignant cells to become invasive and MMPs facilitate their migration to other organs. MMP-3 and MMP-10 are both able to contribute to EMT [9].

Studies concerning the expression of MMP-3 and MMP-10 in BC tissue are found in the available literature [10]. However, to the best of our knowledge, there are no literature reports regarding plasma concentrations of these MMPs in combination with cancer antigen 15-3 (CA 15-3) in patients with BC. Therefore, the aim of the present study was to determine plasma concentrations of MMP-3 and MMP-10, and concentrations of CA 15-3 which is commonly used to detect disease recurrence and monitor treatment efficacy. Concentrations of these markers were assessed at various stages of cancer development and diagnostic criteria were defined based on the analysis of each of the tested parameters independently as well as sets of parameters.

## 2. Materials and Methods

### 2.1. Human Subjects

Table 1 shows the study and control groups. The study group was comprised of 120 patients with BC who were diagnosed at the Department of Oncology, Medical University of Bialystok, Poland, between 2015 and 2018. The cancer stage was determined according to the International Union Against Cancer Tumor-Node-Metastasis (UICC-TNM) Classification. The histopathology of BC was determined in all cases by biopsy of mammary tumor tissue. A complete medical history was obtained from all patients, i.e., a family history of cancer, reproductive history, significant past diseases, hospitalizations, surgeries, medication history, and social history. Tests and procedures performed prior to study commencement included blood sample analysis and a physical examination. Breast mammography and ultrasound scanning, breast core biopsy, and chest X-rays were performed. If necessary, examination of bone marrow, radioisotope bone scans, or brain and chest tomography scans were performed.

The control group was divided into two groups, i.e., 40 patients with benign breast lesions and 40 healthy individuals. All subjects in the control group underwent a breast examination involving palpation and had an ultrasound scan. Benign lesions in the mammary gland were confirmed by histopathological examination. Women with comorbidity or inflammation were excluded from the study to avoid interference with other pathologies.

The study was approved by the Bioethics Committee of Bialystok Medical University (R-I-002/51/2015). All subjects provided written informed consent for study participation.

### 2.2. Plasma Collection and Storage

Venous blood samples were collected from all study participants. Blood was collected in EDTA tubes (S-Monovette, SARSTEDT, Numbrecht, Germany). Plasma samples were centrifuged for 15 min at 1000× *g* to obtain plasma samples and stored at −85 °C until assayed.

### 2.3. Measurement of MMP-3, MMP-10, and CA 15-3

Concentrations of studied stromelysins were measured with the enzyme linked immunosorbent assay (ELISA). Concentrations of MMP-3 were measured using the R&D systems (Abingdon, UK) and concentrations of MMP-10 were measured using EIAab Science (Wuhan, China), according to the manufacturer’s instructions. The manufacturers both stated that a polyclonal antibody specific for human MMP-3 and MMP-10 was precoated onto a microplate. Quantitative determination of CA 15-3 in plasma was conducted by chemiluminescent microparticle immunoassay (CMIA) (Abbott, Chicago, IL, USA), according to the manufacturer’s instructions. The manufacturer states that CA 15-3 test values are defined using 115D8 and DF3 monoclonal antibodies. The CMIA method is predominantly used as an aid in the management of patients with BC and assay values are applied in conjunction with other clinical methods for monitoring BC. The intra-assay coefficient of variation (CV%) of MMP-3 was reported to be 6.1% at a mean concentration of 3.21 ng/mL, SD = 0.197 and MMP-10 was reported to be <3.4%. CA 15-3 was reported to be 2.2% at a mean concentration of 27.0 U/mL, SD = 0.6. The inter-assay coefficient of variation (CV%) of MMP-3 was reported to be 7.0% at a mean concentration of 3.08 ng/mL, SD = 0.217 and MMP-10 was reported to be <7.5%. CA 15-3 was reported to be 2.6% at a mean concentration of 27.0 U/mL, SD = 0.7.

### 2.4. Statistical Analysis

Statistical analysis was performed using the STATISTICA 12.0 program (StatSoft, Tulsa, OK, USA). The Shapiro–Wilk test revealed that the obtained data did not follow a normal distribution. The Mann–Whitney U test, the Kruskal–Wallis test, and a multivariate analysis of various data by the post hoc Dwass–Steel–Critchlow–Fligner analysis revealed significant differences among the groups. The *p* < 0.05 value was considered to be statistically significant. Diagnostic sensitivity (SE) and specificity (SP), predictive value of a positive test result (PPV), and predictive value of a negative test result (NPV) were assessed. The cut-off values were based on the 95th percentile. The receiver operating characteristic (ROC) curves were created using the GraphRoc program for Windows (Windows, Royal, AR, USA). The control group included a group of healthy individuals and a group of subjects with benign breast tumor.

## 3. Results

Table 2 presents plasma levels of all tested parameters. Plasma levels of MMP-10 were significantly elevated in patients with BC as compared with the healthy group and the benign breast tumor group (*p* < 0.001). Moreover, median levels of MMP-10 in all stages of BC were significantly increased as compared with healthy and benign breast tumor subjects (*p* < 0.001). In addition, plasma levels of CA 15-3 were significantly elevated in the BC group (stage III + IV, total BC group) as compared with healthy individuals and benign breast tumor subjects (*p* = 0.002 and *p* = 0.003, respectively).

Median levels of MMP-10, MMP-3, and CA 15-3 in patients with stages III and IV BC were significantly elevated as compared with patients with stage I of the disease (*p* = 0.019, *p* = 0.018, and *p* = 0.003, respectively). Moreover, median levels of MMP-10 and CA 15-3 were significantly higher in patients with stages III + IV BC as compared with those with stage II BC (*p* = 0.025 and *p* = 0.043, respectively).

Concentrations of MMP-10 and CA 15-3 were significantly elevated in the total group of patients with BC as compared with the total control group (*p* < 0.001). Plasma levels of MMP-10 were elevated in all stages of BC as compared with the control group (*p* < 0.001 in every case). Concentrations of CA 15-3 were significantly increased in stages III and IV BC as compared with the total control group (*p* < 0.001).

Table 3 presents the diagnostic criteria, i.e., SE, SP, PPV, NPV in patients with BC. SE for all tested parameters increased in line with disease stage. The highest SE value in the total BC group was demonstrated for MMP-10 (71.7%). Furthermore, SE increased in line with disease progression. The highest values of SE were observed for MMP-10 in all stages of BC (stage I 60.0%, stage II 67.5%, stage III and IV 87.5%).

An increase in diagnostic sensitivity was observed in the combined analysis of tested parameters and CA 15-3. SE for the combination of MMP-10 + CA 15-3 in all stages of the disease (stage I 60.0%, stage II 75.0%, stage III + IV 90.0%) was higher than SE for the combination of MMP-3 + CA 15-3 (stage I 22.5%, Stage II 37.5%, stage III + IV 65.0%). For the combination of MMP-3 + MMP-10 + CA 15-3, the SE value increased to 70.0% (stage I), 82.5% (stage II), and 92.5% (stages III + IV). For the total BC group, SE values were higher for the combination of MMP-3 + MMP-10 + CA 15-3 (81.7%) as compared with the combination of MMP-3 + CA 15-3 (41.7%) and MMP-10 + CA 15-3 (75.0%).

The SP for all tested parameters was the same (95.0%). In the combination of the tested parameters and CA 15-3 (MMP-3 + CA 15-3 and MMP-10 + CA 15-3), SP values were also the same (90.0%). The combination of MMP-3 + MMP-10 + CA 15-3 demonstrated an SP value equaling 85.0%.

PPV in all stages of the disease among all tested parameters was highest for MMP-10 (stage I 92.3%, stage II 93.1%, stage III + IV 94.6%, total group 97.7%). PPV values were higher for CA 15-3 (stage I 71.4%, stage II 84.6%, stage III + IV 92.0%) as compared with MMP-3 in all stages of the disease (stage I 66.7%, stage II 77.8%, stage III + IV 84.6%). Moreover, in the total BC group, the PPV value was higher for MMP-10 (97.7%) as compared with MMP-3 and CA 15-3 (91.7% and 95.1%, respectively). We did not observe higher PPV values for the combination of MMP-10 + CA 15-3 or MMP-3 + CA 15-3 than for single parameters.

NPV in all stages of the disease among all tested parameters was highest for MMP-10 (stage I 70.4%, stage II 74.5%, stage III + IV 88.4%). NPV values were higher for CA 15-3 (stage I 52.1%, stage II 56.7%, stage III + IV 69.1%) than for MMP-3 in all stages of the disease (stage I 51.3%, stage II 53.5%, stage III + IV 56.7%). In the total BC group, the NPV value was higher for MMP-10 (52.8%) than for MMP-3 and CA 15-3 (27.9% and 31.9%, respectively). NPV for the combination of MMP-10 + CA 15-3 in all stages of the disease (stage I 69.2%, stage II 78.3%, stage III + IV 90.0%, total group 54.5%) was higher than NPV for the combination of MMP-3 + CA 15-3 (stage I 53.7%, stage II 59.0%, stage III + IV 72.0%, total group 34%). It was demonstrated that the highest NPV was obtained for the combination of MMP-3 + MMP-10 + CA 15-3 in all stages of BC and in the total BC group (stage I 73.9%, stage II 82.9%, stage III + IV–91.9%, total group 60.7%).

Table 4 presents the area under the curve (AUC) values which denote the potential clinical usefulness and diagnostic power of a diagnostic marker. In the entire group of patients with BC, the areas under the ROC curve were significantly higher as compared with AUC = 0.5 (*p* < 0.001) for all markers used separately and in combination, except for MMP-3 (*p* = 0.7045).

Analysis of single markers revealed that MMP-10 had the highest AUC (0.9166). Moreover, CA 15-3 had a higher AUC value as compared with MMP-3 (0.6743 and 0.5156, respectively). At the earliest stage of the disease, only MMP-10 had a significantly higher AUC (AUC = 0.8692, *p* < 0.001) among single markers. The AUC values for MMP-3 and CA 15-3 did not reach statistical significance (*p* = 0.0713 and *p* = 0.0755, respectively) as compared with AUC = 0.5.

**Figure 1:**
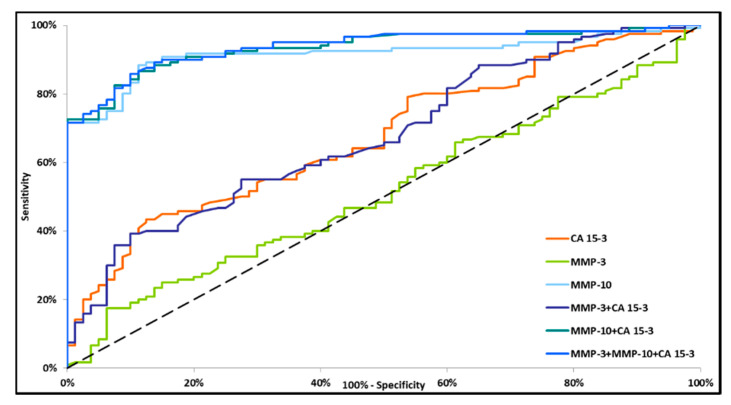
Diagnostic criteria of the ROC curve in total BC group. CA 15-3—cancer antigen 15-3; MMP-3—stromelysin-1; MMP-10—stromelysin-2.

Increasing values of diagnostic power were observed in line with disease progression and in the combined analysis of tested parameters with CA 15-3. Interestingly, the AUC for CA 15-3 and the AUC for the combination of MMP-3 + CA 15-3 were comparable (0.6743 and 0.6765, respectively) (Figure 1). However, among sets of markers, all combinations were found to have significantly larger areas under the ROC curve as compared with AUC = 0.5.

**Figure 2:**
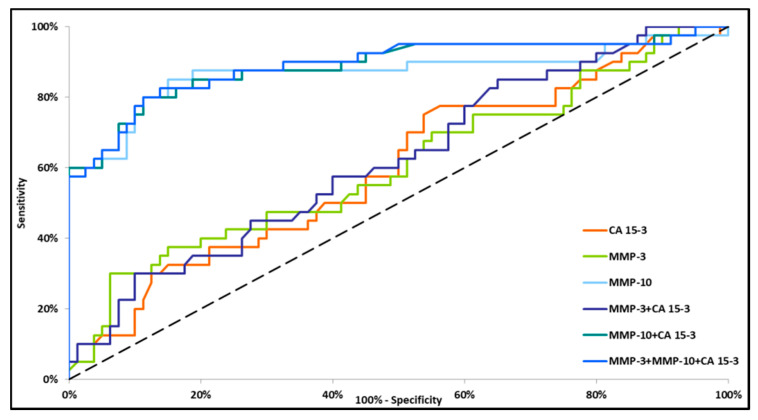
Diagnostic criteria of the ROC curve in stage I of BC. CA 15-3—cancer antigen 15-3; MMP-3—stromelysin-1; MMP-10—stromelysin-2.

**Figure 3:**
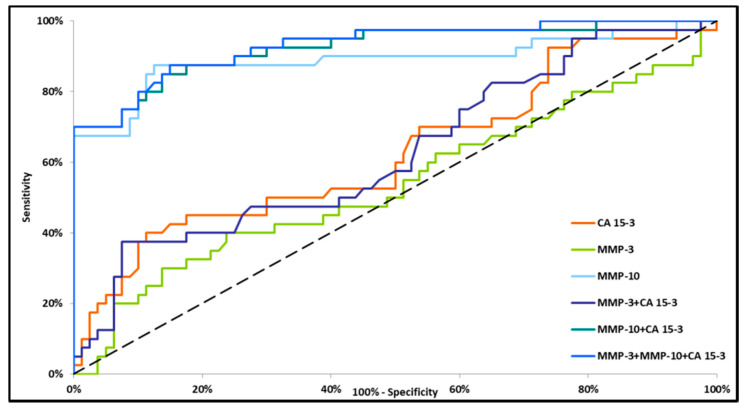
Diagnostic criteria of the ROC curve in stage II of BC. CA 15-3—cancer antigen 15-3; MMP-3—stromelysin-1; MMP-10—stromelysin-2.

**Figure 4:**
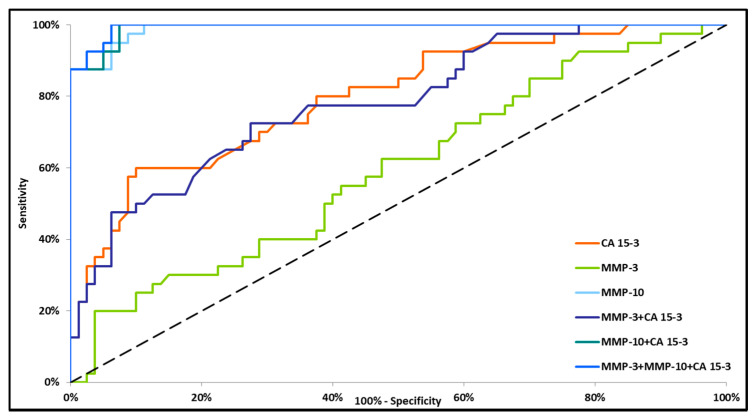
Diagnostic criteria of the ROC curve in stages III and IV BC. CA 15-3—cancer antigen 15-3; MMP-3—stromelysin-1; MMP-10—stromelysin-2.

Small differences in the AUC between the combinations of MMP-10 + CA 15-3 and MMP-3 + MMP-10 + CA 15-3 (0.9349 and 0.9386, respectively) were demonstrated in the total BC group. The highest AUC was observed for MMP-10 + CA 15-3 and MMP-3 + MMP-10 + CA 15-3 (0.8884 and 0.8906, respectively, *p* < 0.001) in stage I BC. The lowest AUC value was observed for the combination of MMP-3 + CA 15-3 (0.6208, *p* = 0.0253) (Figure 2). In stage II BC, MMP-10 and CA 15-3 had significantly higher areas under the ROC curve (0.8902, *p* < 0.001 and 0.6270, *p* = 0.0252, respectively). However, the AUC for MMP-3 did not reach statistical significance (*p* = 0.5171). The combination of tested parameters revealed the highest AUC for the combination of MMP-3 + MMP-10 + CA 15-3 (0.9308, *p* < 0.001), which was higher than the AUC for the combinations of MMP-3 + CA 15-3 and MMP-10 + CA 15-3 (0.6273, *p* = 0.0216 and 0.9244, *p* < 0.001, respectively) (Figure 3). In stages III and IV BC, all markers (except MMP-3) separately and in combination had significantly greater AUC values as compared with AUC = 0.5 (*p* < 0.001). MMP-10 had a greater AUC than CA 15-3 (0.9903 and 0.7970, respectively). Among the sets of markers, MMP-3 + MMP-10 + CA 15-3 had a marginally higher AUC (0.9944) than the combination of MMP-10 + CA 15-3 (0.9919). The lowest AUC was demonstrated for the combination of MMP-3 + CA 15-3 (0.7813) (Figure 4).

## 4. Discussion

Several studies have indicated an important role of MMP-3 and MMP-10 in the stimulation of tumor growth, metastasis, and angiogenesis in BC [11,12]. In the present paper, the areas under the ROC curve were evaluated in order to determine the diagnostic utility of MMP-3 and MMP-10 used separately and in combination with the commonly used BC tumor marker (CA 15-3). To the best of our knowledge, our study is the first to analyze plasma concentrations of stromelysins separately and in combination with CA 15-3 as potential diagnostic markers in BC. There are numerous reports in the available literature that have examined the expression of MMP-3 and MMP-10 in BC tissue, but there was scarce data regarding plasma levels of these enzymes in patients with BC. However, studies on plasma levels of the enzymes in other diseases, including systemic sclerosis [13], type 1 diabetes [14], sepsis [15], or prostate cancer [16] have been published.

Kuźnik-Trocha et al. compared plasma levels of MMP-3 and MMP-10 in patients with systemic sclerosis in whom significantly decreased plasma levels of MMP-3 were observed as compared with healthy subjects (*p* < 0.00001) [13]. However, no differences in MMP-10 concentrations were demonstrated between patients with systemic sclerotic and healthy individuals (*p* > 0.05). The decreased MMP-3 levels may result from the increased activity of tissue inhibitors of matrix metalloproteinases (TIMPs). The authors indicated that higher concentrations of TIMPs in patients with systemic sclerosis as compared with healthy controls were associated with the inhibition of MMP-3 production [13].

Peeters et al. investigated the association among plasma levels of MMPs, including MMP-3 and MMP-10 and cardiovascular disease (CVD) or microvascular complications in patients with type 1 diabetes [14]. The authors hypothesized that abnormal ECM remodeling associated with the action of MMPs may contribute to vascular complications in patients with type 1 diabetes. They also assessed the relationships among enzyme concentrations, and low-grade inflammation (LGI) and endothelial dysfunction (ED). The authors analyzed specimens from 493 patients with type 1 diabetes. Samples from patients with CVD, albuminuria, or retinopathy were separated from the remaining samples. Patients with CVD did not display significantly higher levels of MMPs as compared with patients without CVD. Significantly elevated levels of MMPs, including MMP-3 and MMP-10, were associated with albuminuria. In patients with retinopathy, enhanced levels of another MMP, i.e., MMP-2 were observed. Nevertheless, the study indicated an association among MMP-3 and MMP-10, and macrovascular and microvascular complications in patients with type 1 diabetes. However, the relationships among the complications and the concentrations of MMPs were largely independent of LGI and ED [14].

Elevated levels of MMP-3 have also been observed in patients with sepsis [15,17]. It has been demonstrated that MMPs modulated by different single nucleotide polymorphisms play a critical role in the development of sepsis. Patients with the MMP-3 genotype (*-1612 5A/6A*) had significantly elevated plasma concentrations of MMP-3 as compared with those with the *5A5A* genotype and with the healthy controls. Furthermore, it was found that MMP-3 levels decreased gradually in line with the progression of sepsis [15]. Similar results were obtained by Yazdan-Ashoori et al. who found significantly elevated plasma levels of MMP-3 in patients with severe sepsis [17]. All the diseases mentioned above displayed, in most cases, elevated concentrations of MMP-3 and MMP-10. Enhanced concentrations of MMP-3 and MMP-10 in disease appear to be a response to inflammation. The papers described above demonstrated the pivotal role of stromelysins in various diseases. It can also be assumed that these enzymes could influence the progression of mammary gland tumors.

Lein et al. investigated the concentration of, inter alia, MMP-3 in prostate cancer [16]. Levels of MMP-3 were assessed in patients with metastatic and non-metastatic prostate cancer, patients with benign prostate hyperplasia, and healthy males. Mean concentrations of MMP-3 were significantly higher in patients with metastatic prostate cancer as compared with those with non-metastatic cancer, patients with benign prostate hyperplasia, and healthy controls [16]. The present study also demonstrated significantly elevated plasma levels of MMP-3 in patients with BC, particularly those with stage III or IV of the disease. Aroner et al. investigated plasma concentrations of, inter alia, MMP-3 and BC risk in a prospective control study and found no association between MMP-3 levels and BC risk [18]. The results did not vary significantly with respect to the time since blood samples were obtained, body mass index, or current postmenopausal hormone use. No significant correlations were observed by breast cancer subtypes examined (estrogen receptor negative or estrogen receptor positive cancers). Furthermore, no significant correlations among MMP-3 levels and tumor size or lymph node involvement were observed [18]. A nested case-control study by Kim et al. provided evidence that higher levels of MMP-3 could be related to the risk of distant metastasis in patients with BC [19]. However, the results lacked statistical significance. As in the study by Aroner et al., the authors did not observe any statistical significance between tumor size, lymph node metastases, and estrogen receptor status. In contrast to our findings, no significant correlations were established between tumor stage and plasma levels of MMP-3 [19]. Our study demonstrated that plasma concentrations of both stromelysins were elevated in patients with BC as compared with the control group (patients with benign lesions and healthy subjects). MMP-3 levels were significantly elevated in stages III and IV of the disease; in early stages of BC, the concentrations of MMP-3 were not statistically significant. Interestingly, MMP-10 was significantly elevated in all stages of the disease. Vasaturo et al. assessed plasma concentrations of MMP-3 prior to and following a surgical intervention in patients with adenocarcinomas and fibroadenomas of the mammary gland, and healthy individuals [20]. The authors did not find any statistically significant differences in MMP-3 levels before and after surgery, and they did not establish any significant differences between plasma concentrations of MMP-3 in carcinomas and fibroadenomas [20]. In the present study, we investigated benign breast tumors as one group (adenomas and fibroadenomas), and therefore we were unable to compare patients with adenocarcinomas and those with fibroadenomas. However, our results demonstrated statistically significant differences only between patients with stage III and IV adenocarcinoma and patients with stage I cancer.

Recently, new approaches for non-invasive biomarkers of BC such as circulating carcinoma antigens, circulating tumor cells, circulating cell-free tumor nucleic acids (DNA or RNA), circulating microRNAs, and circulating extracellular vesicles in a variety of body fluids have been introduced to supplement early detection [21]. Several studies have indicated an important role of cell-free DNA (cfDNA) as a new promising detection and monitoring method in BC patients [22,23,24]. The genome of cfDNA originated from primary tumors or metastases contains a genetic alteration that might serve as a potential diagnostic factor. Specific patterns of cfDNA can be detected in plasma or serum. The concentration of cfDNA was significantly lower in healthy patients as compared with those with malignant diseases, as a result of active degradation by nuclease. Moreover, patients with cancer disease had increased cfDNA levels in serum correlated with the incidence of death and the ineffectiveness of pharmacotherapy [22]. However, a study by Peled et al. showed that the cfDNA level could not be used in suspected patients to discriminate BC [25]. Another study found a high diagnostic value of concentration of cfDNA, of which the sensitivity and specificity reached 87 and 87%, respectively [24]. Harnessing the full potential of cfDNA requires refinement of current cfDNA extraction methods. Diagnostic efficacy of cfDNA was shown to be greater than that of traditional tumor markers, nevertheless, the use of cfDNA as a marker for liquid biopsy still lacks standardization in many aspects. The concentration of tumor cfDNA is often very low, thus, any loss of sampled material will reduce the sensitivity of analyses making detection of the cfDNA challenging [26]. Moreover, cfDNA detection is more expensive and requires more advanced equipment and bioinformatic analysis than simple and routinely used ELISA and CMIA methods.

The present paper is part of a large project focusing on MMPs in gynaecological cancers, including BC, focused mainly on MMP-2 and MMP-9 which have a proven pro-carcinogenic effect in breast tissue [27]. Regarding MMP-2, decreased levels were observed in patients with BC as compared with healthy subjects, which was associated with very low concentrations of MMP-2 in stage I of the disease. Moreover, MMP-2 levels were significantly lower in stage I BC as compared with benign breast tumor group. In addition, the plasma concentration of the enzyme was significantly higher in patients with benign breast tumor than in healthy subjects. In the BC group, MMP-2 in combination with CA 15-3 had a significantly higher area under the ROC curve as compared with AUC = 0.5. The AUC value for MMP-2 did not reach statistical significance [28,29]. Interestingly, MMP-9 concentrations were significantly elevated in stage III and IV BC as compared with healthy controls. No statistical differences were observed between MMP-9 concentrations in the group with benign breast tumor and healthy subjects. Moreover, in the BC group, MMP-9 separately and in combination with CA 15-3 had a significantly higher area under the ROC curve as compared with AUC = 0.5 [30,31]. Moreover, in stages II, III and IV BC, MMP-9 separately and in combination with CA 15-3 had a significantly higher AUC as compared with AUC = 0.5 [30]. In the present paper, MMP-10 separately or in combination with CA 15-3 had a significantly higher area under the ROC curve as compared with AUC = 0.5 in all tested groups (total BC, stage I, stage II and stages III + IV). As for MMP-3, only the combination with CA 15-3 had a significantly higher AUC. Therefore, MMP-3 and MMP-10 may play a pivotal role in the diagnosis of BC and could be potential therapy targets in the future.

## 5. Conclusions

Plasma concentrations of MMP-3 and MMP-10 may be useful diagnostic markers for BC, particularly when combined with CA 15-3. Moreover, the obtained results indicate that MMP-10 could be helpful in differentiating stage I of the disease from benign cancer lesions in the mammary gland.

## Figures and Tables

**Table 1:** Characteristics of studied groups, breast cancer (BC) patients and control groups.

Study Group		Number of Cases
**Examined groups**	Breast cancer patients	120
	Median age (range)	54 (21–85)
	Tumor stage	
	I	40
	II	40
	III + IV	40
	Menopausal status	
	Premenopausal	38
	Postmenopausal	82
Control groups	Benign breast tumor lesions patients	40
	Adenoma	18
	Fibroadenoma	22
	Median age (range)	44 (33–63)
	Menopausal status	
	Premenopausal	17
	Postmenopausal	23
	Healthy women	40
	Median age (range)	46 (25–64)
	Menopausal status	
	Premenopausal	18
	Postmenopausal	22

**Table 2:** Plasma levels of tested parameters in patients with BC and control groups.

Tested Groups	MMP-3 (ng/mL)	MMP-10 (ng/mL)	CA 15-3 (U/mL)
Breast Cancer (Median, Range)			
Stage I	6.4 (1.8–15.3)	6387.9 (859.8–8558.4) ^a,b,e^	17.2 (6.2–50.3)
Stage II	6.9 (2.7–24.9)	6670.2 (1544.4–8632.4) ^a,b,e^	18.2 (4.4–48.1)
Stage III + IV	8.2 (2.6–21.9) ^c^	7170.1 (4239.8–9655.6) ^a,b,c,d,^	23.9 (8.9–251.0) ^a,b,c,d,e^
Total group	7.0 (1.8–24.9)	6812.4 (859.8–9655.6) ^a,b,e^	19.4 (4.4–251.0) ^a,b,e^
Control Groups (Median, Range)			
Benign breast tumor	7.8 (2.0–44.8)	2563.2 (1013.2–5250.0)	15.0 (5.2–45.4)
Healthy patients	6.7 (1.8–12.7)	2666.3 (1953.2–5250.0)	16.2 (6.7–29.2)
Total group	6.9 (1.8–44.8)	2624.4 (1013.2–5250.0)	15.5 (5.2–45.4)

Notes: ^a^ Statistically significant for patients with BC as compared with healthy women. ^b^ Statistically significant for patients with BC as compared with benign breast tumor group. ^c^ Statistically significant for patients with BC stages III and IV as compared with patients with BC stage I. ^d^ Statistically significant for patients with BC stages III and IV as compared with patients with BC stage II. ^e^ Statistically significant for patients with BC as compared with the total control group. MMP-3—stromelysin-1; MMP-10—stromelysin-2; CA 15-3—cancer antigen 15-3.

**Table 3:** The diagnostic criteria of tested parameters in patients with BC.

Tested Parameters	Diagnostic Criteria (%)	Breast Cancer			
		Stage I	Stage II	Stage III + IV	Total Group
**MMP-3**	SE	10.0	17.5	27.5	18.3
	SP	95.0	95.0	95.0	95.0
	PPV	66.7	77.8	84.6	91.7
	NPV	51.3	53.5	56.7	27.9
MMP-10	SE	60.0	67.5	87.5	71.7
	SP	95.0	95.0	95.0	95.0
	PPV	92.3	93.1	94.6	97.7
	NPV	70.4	74.5	88.4	52.8
CA 15-3	SE	12.5	27.5	57.5	32.5
	SP	95.0	95.0	95.0	95.0
	PPV	71.4	84.6	92.0	95.1
	NPV	52.1	56.7	69.1	31.9
MMP-3 + CA 15-3	SE	22.5	37.5	65.0	41.7
	SP	90.0	90.0	90.0	90.0
	PPV	69.2	78.9	86.7	92.6
	NPV	53.7	59.0	72.0	34.0
MMP-10 + CA 15-3	SE	60.0	75.0	90.0	75.0
	SP	90.0	90.0	90.0	90.0
	PPV	85.7	88.2	90.0	95.7
	NPV	69.2	78.3	90.0	54.5
MMP-3 + MMP-10 + CA 15-3	SE	70.0	82.5	92.5	81.7
	SP	85.0	85.0	85.0	85.0
	PPV	82.4	84.6	86.0	94.2
	NPV	73.9	82.9	91.9	60.7

MMP-3—stromelysin-1; MMP-10—stromelysin-2; CA 15-3—cancer antigen 15-3; SE—sensitivity; SP—specificity; PPV—positive test result; NPV—negative test result.

**Table 4:** Diagnostic criteria of the receiver operating characteristic (ROC) curve for tested parameters in BC.

Tested Parameters	AUC	SE	95% C.I. (AUC)	*p* (AUC = 0.5)
ROC Criteria in Breast Cancer (total group)				
MMP-3	0.5156	0.0412	(0.435–0.596)	0.7045
MMP-10	0.9166	0.0210	(0.875–0.958)	<0.001
CA 15-3	0.6743	0.0378	(0.600–0.748)	<0.001
MMP-3 + CA 15-3	0.6765	0.0380	(0.602–0.751)	<0.001
MMP-10 + CA 15-3	0.9349	0.0171	(0.901–0.968)	<0.001
MMP-3 + MMP-10 + CA 15-3	0.9386	0.0165	(0.906–0.971)	<0.001
ROC Criteria in Breast Cancer (I stage)				
MMP-3	0.6031	0.0572	(0.491–0.715)	0.0713
MMP-10	0.8692	0.0437	(0.784–0.955)	<0.001
CA 15-3	0.5988	0.0556	(0.490–0.708)	0.0755
MMP-3 + CA 15-3	0.6208	0.0540	(0.515–0.727)	0.0253
MMP-10 + CA 15-3	0.8884	0.0375	(0.815–0.962)	<0.001
MMP-3 + MMP-10 + CA 15-3	0.8906	0.0373	(0.818–0.964)	<0.001
ROC Criteria in Breast Cancer (II stage)				
MMP-3	0.5386	0.0596	(0.422–0.655)	0.5171
MMP-10	0.8902	0.0396	(0.813–0.968)	<0.001
CA 15-3	0.6270	0.0568	(0.516–0.738)	0.0252
MMP-3 + CA 15-3	0.6273	0.0554	(0.519–0.736)	0.0216
MMP-10 + CA 15-3	0.9244	0.0277	(0.870–0.979)	<0.001
MMP-3 + MMP-10 + CA 15-3	0.9308	0.0254	(0.881–0.981)	<0.001
ROC Criteria in Breast Cancer (III + IV Stage)				
MMP-3	0.5948	0.0550	(0.487–0.703)	0.0848
MMP-10	0.9903	0.0055	(0.980–1.001)	<0.001
CA 15-3	0.7970	0.0433	(0.712–0.882)	<0.001
MMP-3 + CA 15-3	0.7813	0.0445	(0.694–0.868)	<0.001
MMP-10 + CA 15-3	0.9919	0.0048	(0.982–1.001)	<0.001
MMP-3 + MMP-10 + CA 15-3	0.9944	0.0036	(0.987–1.002)	<0.001

MMP-3—stromelysin-1; MMP-10—stromelysin-2; CA 15-3—cancer antigen 15-3; ROC—the receiver operating characteristic; AUC—the area under the curve; SE—sensitivity; 95% C.I.—95% confidence interval.
